# A complex heterozygous mutation in *PADI6* causes early embryo arrest: A case report

**DOI:** 10.3389/fgene.2022.1104085

**Published:** 2023-01-10

**Authors:** Ting Zhang, Peng Liu, Guanfeng Yao, Xin Zhang, Cuijuan Cao

**Affiliations:** Hebei Key Laboratory of Reproductive Medicine, Department of Reproductive Medicine, Hebei Reproductive Hospital, Shijiazhuang, China

**Keywords:** complex heterozygous mutation, case report, early embryo arrest, PADI6 gene, female infertility

## Abstract

**Background:** The *PADI6* gene is a component of the subcortical maternal effect complex (SCMC). Mutations in the *PADI6* gene, which was the first gene discovered to impact the activation process of the human embryonic genome, have been shown to induce early embryo arrest.

**Case:** A 29-year-old lady with primary infertility underwent *in vitro* fertilization embryo transfer (IVF-ET) for tubal reasons, who had normal hormone levels and ovarian reserve. A Progestin-Primed Ovarian Stimulation (PPOS) protocol of Ovarian stimulation with IVF was performed. The total of Gonadotropin (Gn) stimulation with u-FSH was 2100 IU, which lasted for 10 days. When three follicles measuring less than 18 mm in diameter were seen, r-hCG 250 ug and triptorelin acetate 0.2 mg were injected to trigger oocyte maturation. Nineteen oocytes (including thirteen MII oocytes) were picked up 37 h after the trigger, and seven of these were normal fertilized. Unfortunately, these many embryos were stopped at the 1- or 2-cell stage, hence this infertile patient’s IVF treatment won't result in an embryo transfer. Using whole-exome sequencing, a complex heterozygous mutation in *PADI6* was discovered: c. 1247T>C [p.Ile416Thr] in exon 12 of *PADI6*, and c. 2009_2010del [p.Glu670GlyfsTer48] in exon 17 of *PADI6*.

**Conclusion:** We found a complex heterozygous mutation in the *PADI6* gene (c. 1247T>C; c. 2009_2010del) that caused embryos were arrested at the 1- or 2- cell stage. The discovery in this patient adds to the evidence showing the PADI6 gene mutation causes early embryo arrest in humans.

## Introduction

The subcortical maternal complex (SCMC) is an oocyte-to-embryo-specific maternal functional module that is important for embryo development in both mice and humans (Li et al., 2008; Zhu et al., 2015). In addition to the multiple effects that the SCMC was thought to have during oocyte maturation and early embryo development in oogenesis (Bebbere and Albertini, 2021). The complex was connected to essential events that contribute to the transition from oocyte to embryo, such as meiotic spindle formation and placement, translation regulating, organelle redistribution, and epigenetic reprogramming (Bebbere and Albertini, 2021).

Genes encoding SCMC members that contribute to pre-implantation embryonic arrest and regulate accurate zygote cleavage have been identified (Zheng et al., 2021). *PADI6*, *TLE6*, *KHDC3L*, *NLRP2*, *NLRP5*, and *NLRP7* mutations in SCMC have all been provided a link to abnormal fertilization or embryo arrest (Wang et al., 2018; Mu et al., 2019; Maddirevula et al., 2020; Xu et al., 2020; Zheng et al., 2021). Early identification of causal genetic variations in the field of reproductive medicine can enhance the diagnosis of unexplained infertility, allowing for enhanced treatment and advice.

## Case presentation

A 29-year-old woman suffered from primary infertility who had been married for 2 years. The woman’s physical condition was normal, and no abnormalities were discovered during the medical examination. She also had regular menstrual periods that lasted 28–30 days (menarche occurred at 13 years old). Salpingography indicated that the left fallopian tube was unobstructed and that the right fallopian tube was partially obstructed. She then tried natural pregnancy for 7 cycles, monitoring follicle growth with B-ultrasonography in a reproductive center. Mature follicles were exhausted in all seven cycles (sometimes on the left, sometimes on the right), but no pregnancy occurred. The patient’s husband was married for the second time, and he had a healthy child from his previous marriage. To get her pregnant, we tried to approach *in vitro* fertilization embryo transfer (IVF-ET).

**FIGURE 1:**
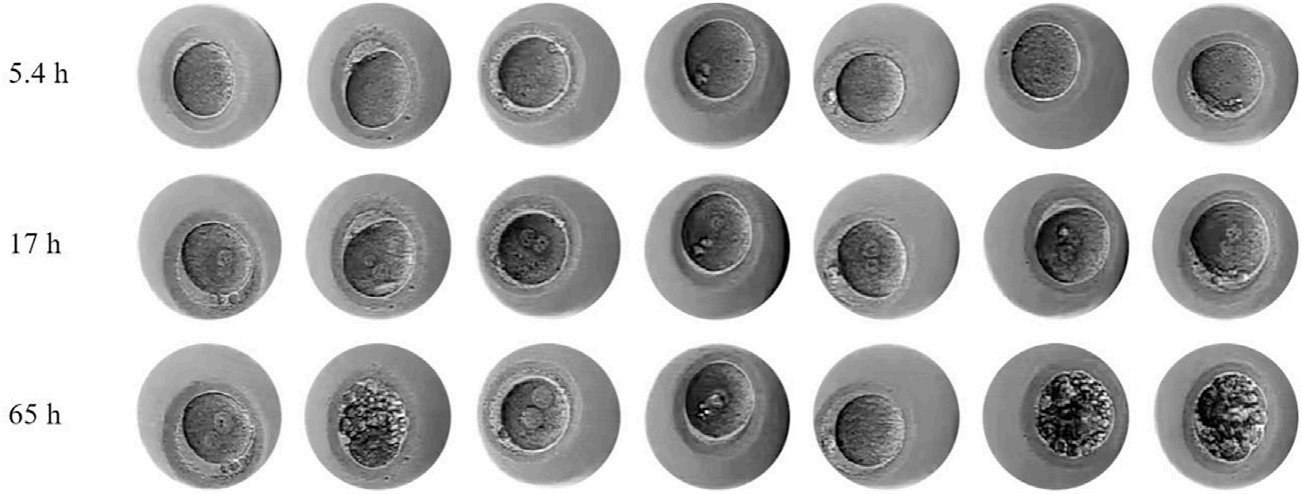
Morphological characteristics of seven embryonic observed by time-lapse imaging.

An ovarian stimulation treatment using the progestin-primed ovarian stimulation (PPOS) protocol was given on the patient, who had normal hormone levels and ovarian reserve (Table 1). Gonadotropin (Gn) stimulation with urinary follicle stimulating hormone (u-FSH) (Lishenbao®, LIVZON, Zhuhai, China) was started at 225 IU/day, and the total of Gn was 2100 IU for 10 days. Up until the trigger day, oral progesterone (Dupbaston®, Abbott Biologicals B.V, Netherlands) 20 mg was used daily. When three 18 mm diameter follicles were observed, recombinant human chorionic gonadotropin (r-hCG) (Ovidrel®, Merk-Serono, Spain) 250 ug and triptorelin acetate injection (Decapeptyl®, Ferring AG, Switzerland) 0.2 mg were used to stimulate oocyte maturation. Nineteen oocytes, including thirteen MII oocytes, were pick up by transvaginal ultrasound guidance 37 h after the trigger. At 4 h after retrieval of oocytes insemination was performed by normally IVF, and fertilized embryos were cultured in a time-lapse incubator. Light microscopy and time-lapse microscopy were used to determine the morphology of embryos at various growth stages during 4–5 h of IVF. Seven of them were normally fertilized, and all embryos were arrested at the 1- or 2-cell stage following culture (Figure 1). Embryo grading and viable embryo definition followed the previously documented procedure (Alpha Scientists in Reproductive Medicine and ESHRE Special Interest Group of Embryology, 2011; ESHRE Special Interest Group of Embryology and Alpha Scientists in Reproductive Medicine, 2017).

**TABLE 1:** Sex hormone level and ovarian reserve of the patient.

Day	FSH (IU/L)	LH (IU/L)	E_2_ (pg/ml)	P (ng/ml)	AMH (ng/ml)	AFC/Follicles
MC2	7.98	5.22	34.92	0.42	2.5	16
Trigger day	NA	5.16	3334.19	1.45	NA	15

MC2, menstrual cycle day 2; FSH, follicle stimulating hormone; LH, luteinizing hormone; E_2_, estrogen; P, progesterone; NA, not available. The follicles on the day of triggering were follicles with diameter ≥12 mm.

**FIGURE 2:**
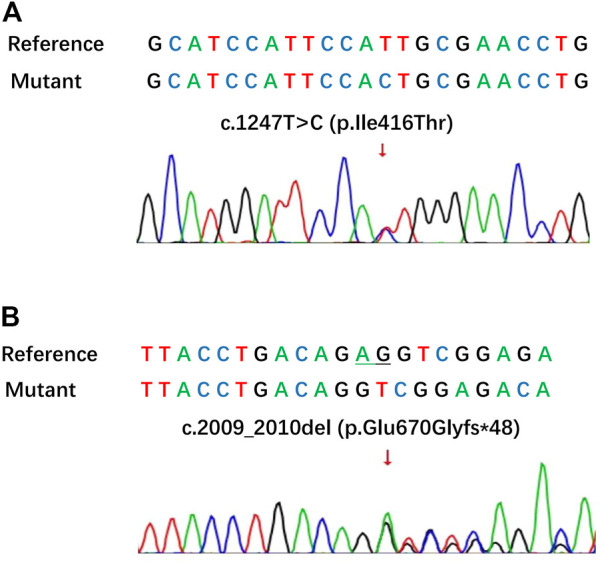
A novel complex heterozygous mutation in *PADI6* of the patient. **(A)** The missense mutation of **(C)** 1247T>C in the *PADI6* gene; **(B)** The frameshift mutation of **(C)** 2009_2010del in the *PADI6* gene.

In this infertile woman, IVF failed in no embryo transfer and early embryo arrest. Then, for the infertile woman, a novel complex heterozygous mutation in *PADI6* was found using whole-exome sequencing (WES). Singer sequencing was used for verification, and the results were as follows: c. 1247T>C [p.Ile416Thr] in exon 12 of *PADI6* (Figures 2A,C). 2009_2010del [p.Glu670GlyfsTer48] in exon 17 of *PADI6* (Figure 2B).

## Discussion

In this patient, all embryos arrest at the 1- or 2-cell stage on the second day after fertilization, resulting in the failure of IVF assisted reproductive treatment. Early embryo arrest occurs when the development of preimplantation embryos stops, and embryos at the cleavage stage with development retardation are unable to form blastocysts, which is one of the causes for the failure of assisted reproduction (Wang et al., 2020). Normal preimplantation embryonic development is an essential step in improving a successful pregnancy. On day 3 of culture, the zygote experiences three consecutive episodes of cytokinesis and forms six to eight blastomeres (ESHRE Special Interest Group of Embryology and Alpha Scientists in Reproductive Medicine, 2017). Whenever all embryos in a patient’s infertility suffer from developmental arrest, the patient’s IVF/intracytoplasmic sperm injection (ICSI) cycle fails.

Several recent studies had identified maternal-effect factors that were critical in the development of preimplantation embryos (Wang et al., 2018). Normal embryonic development depended on maternal-effect genes to coordinate the oocyte-to-embryo transition, and the SCMC was a maternal functional module that was particular to the oocyte-to-embryo transition (Bebbere and Albertini, 2021). The SCMC, which was situated in the subcortex and consisted of maternal functional modules specific to the oocyte to the embryo, was important for embryonic development in both mice and humans; altering SCMC genes in mice and humans led to embryonic arrest (Wang et al., 2018; Bebbere and Albertini, 2021). *PADI6* (peptidyl arginine deiminase, type VI), and *TLE6* (transducin-like enhancer of split 6) mutations, for instance, induced embryonic arrest before the 8-cell stage (Bebbere and Albertini, 2021; Zheng et al., 2021).

The *PADI* family was made up of five members: *PADI1, PADI2, PADI3, PADI4*, and *PADI6* (Liu et al., 2021). *PADI6* was only found in oocytes and early embryos, and it was required for oocyte maturation and embryo development (Liu et al., 2021), and was one of the SCMC components that localized to other components in oocytes and early embryos (Xu et al., 2016; Wang et al., 2022). *PADI6* encoded a peptidylalarginine deiminase, the amino acid sequence of which was substantially conserved across mammalian peptidylarginine deiminases (Xu et al., 2016; Lu et al., 2017). The stored *PADI6* mRNAs were mainly destroyed in the zygote (Li et al., 2008; Yurttas et al., 2008), with spindle microtubule construction and mobility changing to mediate correct cleavage in early embryo development (Yurttas et al., 2008; Kan et al., 2011). Mutation in the *PADI6* gene Because oocytes lack a particular cytoskeletal structure known as cytoplasmic lattices, they exhibit abnormal organelle placement and redistribution (Liu et al., 2017). *PADI6* played an important evolutionary and developmental role in the early embryonic development of advanced vertebrate species (Liu et al., 2017).

In 2016, Xu et al. (2016) first reported that *PADI6* gene mutation was associated with embryonic arrest due to embryonic genome activation failure in human. *PADI6* was considered to encode a protein involved in the SCMC, which was required for embryonic advancement beyond the 2-cell stage (Li et al., 2010). The homozygous mutations and the compound-heterozygous mutations of *PADI6* have been reported to cause embryo arrest (Wang et al., 2022; Xu et al., 2022). In this case, we found a missense mutation (c. 1247T>C) and a frameshift mutation (c. 2009 2010del) in *PADI6* by WES, as well as embryos with aberrant cleavage patterns and embryos at 1- or 2-cell arrest on Day 2. Xu et al. (2016) found that the compound-heterozygous mutation of *PADI6* gene (c. 633T>C; c. 2009_2010del) caused early embryo arrest in human. Dong et al. (2022) found that a compound-heterozygous mutation of *PADI6* gene (c. 1247T>C; c.294 + 5G>A) and a homozygous mutations (c. 1247T>C) caused embryos were arrested at the 2- or 7- cell stage. We considered that the compound heterozygous mutation of *PADI6* gene (c. 1247T>C; c. 2009 2010del) in this patient was the reason of early embryos were all arrested at 1- or 2- cell. The patient’s parents didn’t check the *PADI6* gene mutation because of the patient’s reason. We propose that if a young woman with infertility has no evident risk factors for abnormal embryo cleavage but it occurs, the cause should be aggressively examined, as well as whether there are gene mutations that influence the quality of oocytes or embryos. Although abnormal embryo cleavage induced by gene mutation was uncommon, it did occur in those whose problems could not be explained by conventional understanding.

## Conclusion

We found a complex heterozygous mutation in the *PADI6* gene (c. 1247T>C; c. 2009_2010del) that caused embryos were arrested at the 1- or 2- cell stage. The discovery in this patient adds to the evidence showing the PADI6 gene mutation causes early embryo arrest in humans.

## Data Availability

The original contributions presented in the study are included in the article/Supplementary Material, further inquiries can be directed to the corresponding author.
